# Production of Composts from Cheese Whey and Agro-Livestock and Their Valorization in Volcanic-Ash-Affected Soil Cultivated with *Lactuca sativa* L.

**DOI:** 10.3390/plants15101507

**Published:** 2026-05-15

**Authors:** Steven Ramos-Romero, Irene Gavilanes-Terán, Julio Idrovo-Novillo, Sandra N. Escobar-Arrieta, María José Bermeo, Alessandro Idrovo-Gavilanes, Julio Idrovo-Gavilanes, Ángel A. Carbonell-Barrachina, Antonio J. Signes-Pastor, Concepción Paredes

**Affiliations:** 1 Faculty of Agricultural Industries and Environmental Sciences, Carchi State Polytechnic University, Tulcán 040102, Carchi, Ecuador; steven.ramos@upec.edu.ec; 2 Faculty of Science, Higher Polytechnic School of Chimborazo, Riobamba 060155, Chimborazo, Ecuador; irene.gavilanes@espoch.edu.ec (I.G.-T.); julio.idrovo@espoch.edu.ec (J.I.-N.); saescobar@espoch.edu.ec (S.N.E.-A.); jose.bermeo@espoch.edu.ec (M.J.B.); 3 Faculty of Medicine and Health Sciences, Ghent University, Corneel Heymanslaan 10, 9000 Ghent, Belgium; alessandro.idrovo@ugent.be; 4 Institute for Research in Biomedicine of Lleida (IRBLleida), Av. Alcalde Rovira Roure, 80, 25198 Lleida, Spain; jidrovo@irblleida.cat; 5 Agri-Food and Agro-Environmental Research and Innovation Institute (CIAGRO-UMH), Miguel Hernandez University, EPS-Orihuela, ctra. Beniel km 3.2, 03312 Orihuela, Spain; angel.carbonell@umh.es (Á.A.C.-B.); 6 Alicante Institute for Health and Biomedical Research, Miguel Hernandez University (ISABIAL-UMH), 03010 Alicante, Spain; asignes@umh.es; 7 Nutrition Epidemiology Unit, Miguel Hernandez University, 03550 Sant Joan d’Alacant, Spain; 8 CIBER Epidemiology and Public Health (CIBERESP), Carlos III Health Institute, 28029 Madrid, Spain

**Keywords:** cheese industry wastewater, agricultural and livestock wastes, composting, contaminated volcanic soil, bioremediation, heavy metal uptake, lettuce

## Abstract

The deposition of volcanic ash in areas affected by erupting volcanoes can contaminate the soil with heavy metals, thereby jeopardizing food security and public health. This study focused on the use of compost for the bioremediation of this type of contaminated soil and on evaluating the effectiveness of this remediation technique in a horticultural crop. To this end, composts made from organic waste generated in the areas with volcanic-ash-affected soil, such as crop residues, cow manure, and cheese whey, were used. The design and optimization of the composting process for these wastes were described using three piles with the same proportion of crop residues and cow manure but different doses of whey (pile 1: without whey, pile 2: whey diluted with water (1:2 (*v*:*v*)); and pile 3: with undiluted whey) and by monitoring the evolution of physicochemical and biological parameters throughout the compositing process. The effectiveness of the composts obtained for soil remediation was evaluated by assessing the physiological response of a lettuce crop in pots. Five treatments were used: control soil without fertilization, inorganic fertilization, and the three composts obtained. The main agronomic properties of the soil and heavy metal availability were measured, along with the physiological and chemical parameters of the lettuce, including growth and macronutrient and heavy metal content. The results obtained in the composting experiment showed that the addition of cheese whey only affected the rate of organic matter degradation and the salt content of the final composts, without negatively affecting the stability and humification of their organic matter or their plant nutrient content. In the pot experiment, all composts improved soil fertility and reduced the availability of Ni, As, Cd, and Pb, but this did not consistently reduce uptake into lettuce, except in the case of Pb. Therefore, it is advisable to adjust the compost application rate and optimize crop selection to minimize the impact of heavy metals on the food chain, thereby ensuring safe production.

## 1. Introduction

Ecuador is located in an area of high volcanic activity, currently having up to 15 active volcanoes and two erupting continental volcanoes (El Reventador and Sangay) [1]. Volcanic eruptions in recent decades have deposited large amounts of ash in areas near these volcanoes. Volcanic ash is generally composed of Si, Al, Fe (80%), Na, Ca, Mg, K, and Pb oxides, as well as other heavy metals such as V, Cr, Co, Ni, Zn, As, Cd, and Hg [2]. Therefore, volcanic ash can be considered a multinutrient mineral fertilizer, but it is also a significant source of heavy metal soil contamination. This soil contamination can pose major risks to human health by transferring heavy metals into the food chain, as many rural populations engaged in agricultural and livestock farming activities live near volcanoes, attracted by the soil’s fertility [3]. Various studies have reported the transfer of heavy metals from the soil to agricultural or livestock products in Ecuador. Romero-Estévez et al. [4] showed in a review study that various agricultural products (bananas, cocoa, manioc, tomatoes, etc.) contain amounts of non-essential metals that are above the maximum limits established for food by the FAO. They pointed to the volcanic origin of most Ecuadorian soils as one of the sources of heavy metal contamination. Carrera-Beltrán et al. [5] also found Cd and Pb contents above the limits regulated by national and international standards in potatoes and maize in a study on volcanic ash deposition from the Tungurahua volcano in affected areas of the Pelileo, Quero, Cevallos, and Penipe cantons. Finally, Carrera-Beltrán et al. [6] evaluated heavy metal transfer from soil to forage and milk in the Bilbao parish, located on the slope of the Tungurahua volcano and severely affected by volcanic ash deposition during this volcano’s latest eruption. These authors observed that the forage had high bioaccumulation of Pb, Cd, As, Hg, and Se, but there was limited transfer of these elements to milk, with only high Se concentrations detected.

The phytoavailability of heavy metals in soil depends on the chemical characteristics of each element, which are mainly determined by the soil’s physical, physicochemical, chemical, and biological properties. Soil pH is a major factor influencing heavy metal mobility. This parameter determines the speciation of metals at the soil-solution interface, their solubility, and the adsorption and desorption processes. In acidic soils, the solubility of minerals and hydrous oxides to which heavy metals are adsorbed increases, thus promoting the mobility and solubility of these contaminants. At high pH levels, heavy metals can be immobilized by adsorption onto the surfaces of various minerals or by precipitation as insoluble hydroxides, phosphates, or carbonates [7]. Soil organic matter is another factor that contributes to heavy metal immobilization by providing functional groups such as amine, hydroxyl, and carboxyl, which have a strong affinity for these contaminants. These groups are protonated or deprotonated depending on the pH, altering their charge and influencing the adsorption or desorption of cations from heavy metals [8]. The presence of organic matter, clays, and metal oxides increases the soil’s cation exchange capacity and thus the sorption area of heavy metals [9]. Heavy metal solubility and transport can also be affected by the microbial activity and root exudates [9,10].

High heavy metal concentrations in plants can alter their metabolism, morphology, and biochemical processes. Arsenic can cause significant damage to plants even at low concentrations [11], and Cd decreases plant metabolic activity, causes chlorosis, and changes the absorption rates of other nutrients [12]. Cr modifies photosynthetic efficiency, alters the germination process, and decreases biomass production [12,13]. High concentrations of Pb in plants alter photosynthetic processes, reduce overall plant growth, and decrease seed germination and root elongation [12,14].

Compost application is widely reported as an effective strategy for remediating soils contaminated with heavy metals, as it can reduce the mobility of these pollutants through various mechanisms. Udovic and McBride [15] observed that applying household/garden compost to soil significantly reduced Pb bioavailability. This was due to an increase in soil pH (reducing Pb solubility), as well as added phosphorus compounds (precipitation of Pb-phosphates) and humic materials (formation of stable Pb-humified organic matter complexes). Meier et al. [16] studied the effects of applying chicken manure compost to Cu-contaminated soil. They found that soil pH increased, exchangeable Cu decreased because the amount of Cu bound to organic fractions increased, soil microorganism activity was stimulated, the composition of the microbial community was modified, probably due to reduced Cu availability and increased carbon and nutrient sources for microorganisms, and plant biomass production increased in the soil. Zeng et al. [17] found that applying chicken manure compost to the soil improved soil fertility and mitigated Pb/Cd absorption, although they warned of the risks of Zn/Cu accumulation, requiring careful management of this compost for bioremediation of paddy soil contaminated with heavy metals from mining activities. Liu et al. [18] reported that using chicken manure compost was effective in remediating Cd-contaminated soils. The bioavailability of this heavy metal to a wheat crop was reduced due to increased soil pH, the complexation of Cd by the organic matter, and the precipitation of Cd-phosphorus compounds. Rehman et al. [19] evaluated the effects of compost application on the physiological and biochemical characteristics of maize grown in Ni-contaminated soil. This study concluded that the use of compost reduced the availability of this heavy metal, likely due to the increase in soil pH, the high cation exchange capacity of the compost’s organic matter, and the increased nutrient uptake by the plants, which competed with Ni uptake at the root surface. However, applying compost to the soil can increase As mobility. Mehmood et al. [20] observed that increased soil pH and cation exchange capacity produced by adding compost favored As-oxyanion desorption as a result of reduced positive charges in the soil, thus increasing As mobility. Therefore, special attention should be paid to remediating agricultural soils contaminated with multiple heavy metals coexisting with As through compost to reduce As bioavailability.

The use of compost for the bioremediation of soil contaminated by volcanic ash in Ecuador has been little explored, mainly due to limited resources. However, using compost to remediate agricultural soils contaminated with heavy metals is an economical, highly practical, and environmentally friendly method, as compost can be produced from waste generated near the areas affected by these contaminants. As indicated above, economic activities in areas affected by volcanic ash are essentially agricultural and livestock-related. Therefore, compost can be produced from agro-livestock and associated agro-industry wastes, which can be used to remediate soils contaminated with heavy metals from volcanic ash.

In this study, crop residues (maize and beans) were co-composted with cow dung, and cheese whey was used as a moisture source. Currently, managing these residues is not optimized in Ecuador. Crop residues are generally burned, and manure is applied to the soil without any technical criteria, causing various environmental impacts on the atmosphere, soil, and water [21]. Furthermore, the use of cheese whey is very limited, as only 1.5% of this by-product is reincorporated into the production system, mainly in the manufacture of dairy products such as yogurt, fermented and non-fermented beverages, cream cheese, and fresh cheese [22]. As a result, much of the whey generated in the country is disposed of improperly, either by direct discharge into water bodies or by application to agricultural soils, causing significant environmental damage to these environments [23]. Co-composting agricultural and livestock waste has been widely reported, and various studies have even been conducted in Ecuador. Tomato and broccoli crop residues have been co-composted with chicken manure [24], rose crop residues with manure from different fowls [25], and a mixture of residues from different herbaceous, horticultural, and fruit crops with cow, guinea pig, or chicken manure [21]. However, information on whey treatment using composting processes is still limited. This liquid waste has a high organic load and a significant nutrient content for plants (N, P, and K), as well as low concentrations of heavy metals, characteristics that are suitable for improving the quality of the final compost [23]. Some studies have been conducted on the use of cheese whey as an additive in the composting process to promote microbial activity and provide the necessary moisture content. Michalopoulos et al. [26] studied the combined processes of anaerobic digestion and composting for the joint treatment of different manures and cheese whey. In this study, the composting process was carried out on the solid fraction separated from the initial waste mixture, resulting in adequate development of the composting process and the production of phytotoxicity-free compost. Rajabi Hamedani et al. [27] conducted an environmental and economic analysis of the combined anaerobic digestion and composting process applied to a mixture of manure, plant waste, and cheese whey. The results show that this integrated system generated environmental and economic benefits related to producing biofuels and organic soil fertilizers. Alfonzo et al. [28] produced sanitized and stable compost by co-composting grape pomace, green herbaceous crop, and pruning residues with deproteinized whey to produce stable, high-quality compost. Finally, Pivato et al. [29] co-composted wood chips and biochar, using cheese whey as a source of water-soluble carbon to extract the heat generated during composting through a hydraulic circuit. However, whey also contains high levels of salts, which may limit its use as an additive in the composting of other wastes [23]. This issue has been little explored in the studies found on co-composting whey with other organic wastes. Only Şahin et al. [30] evaluated the effect of applying different amounts of cheese whey to the co-composting of rice husk with sewage sludge or chicken manure on the efficiency of the process and the quality of the final compost.

Based on an analysis of the above information, we hypothesize that co-composting of cheese whey with plant residues and cow dung may be an effective way to utilize these residues by using the resulting composts to reduce the bioavailability of heavy metals in soils affected by volcanic ash, thereby reducing the risk of migration of these metals into plant tissues. Numerous studies on composting cheese whey with other wastes have focused mainly on the technological feasibility of the process and the quality of the final compost; however, limited information is available on the composting of this liquid waste in terms of optimizing the process based on the factors limiting its use. Furthermore, in Ecuador, it is uncommon to use techniques to address soil contamination caused by heavy metals. Therefore, the main objectives of our study were: (i) to optimize co-composting cheese whey with agro-livestock wastes based on the quantity or dilution of this liquid waste to reduce the salinity of the final compost; and (ii) to evaluate the effectiveness of the compost obtained in the bioremediation of soils contaminated by volcanic ash by studying the physical–chemical and chemical properties of the soil and the physiological response of a lettuce crop (*Lactuca sativa* L.). This crop was chosen due to its global consumption and high sensitivity to contamination [31]. The results obtained in this plant and in the soil properties indicate the effectiveness of the bioremediation technique studied.

## 2. Materials and Methods

### 2.1. Composting Experiment

The composting experiment was carried out at the research station of the Higher Polytechnic School of Chimborazo (Riobamba, Ecuador). Three piles of agro-livestock waste were designed with the same composition and varying amounts of cheese whey to optimize the dose of this liquid waste, considering its high salt content. The composition of these piles was as follows (in fresh weight): 60% maize waste + 5% bean waste + 35% cow dung. These waste mixtures were prepared with plant waste crushed to a particle size of <5 cm and mixed manually with cow dung, forming trapezoidal piles with dimensions of 2 × 3 m at the base, a height of 1.5 m, and a weight of 1000 kg. The proportion of the residues within the mixture was selected to obtain an initial total organic carbon (Corg)/total nitrogen (Nt) ratio close to the range of 25–35 (established as adequate for an optimal balance of nutrients for the microorganisms in the process and to reduce losses of nutrients such as nitrogen [32]). These piles were not replicated due to logistical and space constraints. The composting experiment was conducted on a semi-industrial scale (1000 kg of waste mixture), and it was difficult to obtain tons of homogeneous organic material for multiple replicates. Furthermore, the experimental composting facility used had limited space, which did not allow for the simultaneous replication of the tested piles. Throughout the biooxidative phase of composting, water, cheese whey diluted 50% with water, and undiluted cheese whey were added to piles 1, 2, and 3, respectively, to maintain their moisture content within the range of 40–60%. A 1:2 (*v*/*v*) dilution of the whey was chosen to reduce its salinity, but no greater dilutions of this liquid residue were made in order to valorize the greatest possible volume of whey. The total amount added by each source of moisture used was 0.35 L/kg of the initial fresh weight of the waste. The water and cheese whey were added using a sprinkler hose, with the diluted and undiluted whey transferred from separate tanks using a submersible pump. The main characteristics of the initial materials are listed in Tables S1 and S2.

The turned windrow composting system was used. Piles 1 and 2 were turned three times, while pile 3 was only turned twice. The turnings were carried out when the temperature of the piles was below the values corresponding to the thermophilic phase (<40 °C). This was done to provide the oxygen necessary for the aerobic degradation of the organic matter and, at the same time, to homogenize the mixture of waste to be decomposed. The temperatures developed throughout the bio-oxidative phase of composting were measured daily with a portable probe at a depth of 30 cm at five different points in the pile, and the ambient temperature was also measured daily. The bio-oxidative phase was considered complete when no re-heating occurred after turning and the temperature of the piles was close to ambient temperature. This phase lasted approximately 80 days. The piles were then left to mature without ventilation for 30 days. The piles were sampled three times during the biooxidative phase (at the beginning of the process, during the thermophilic phase, and at the end of the biooxidative phase) and at the end of maturation. Subsamples were collected from seven randomly selected points covering the entire profile of the pile. These subsamples were mixed to obtain a representative 2 kg sample, which was dried at 60 °C, ground, and sieved to 0.5 mm for subsequent analysis. All determinations were performed in triplicate.

### 2.2. Pot Experiment

The pot experiment with lettuce (*Lactuca sativa* L.) was conducted in the greenhouse facilities of the Higher Polytechnic School of Chimborazo (Riobamba, Ecuador) from 1 January to 10 March 2024 (70 days). The maximum and minimum ambient temperature and humidity levels recorded during the experimental period were 13.5–35.4 °C and 19–65%, respectively.

The soil used for this study was collected from the surface layer (0–30 cm) at Chambo canton (Chimborazo, Ecuador, 1°44′31″ S; 78°35′19″ W, elevation 2814 m a.s.l.). This area was significantly affected by ashfall from the most recent eruptions of the Sangay volcano (2.002° S; 78.341° W, elevation 5230 m a.s.l. and diameter 10–12 km). The soil in this area has been classified as an Inceptisol [33], with a granulometry of 39 ± 0% silt, 12 ± 0% clay, 49 ± 0% sand, and a loamy texture. The pH and electrical conductivity (EC) values in water extracts were 6.73 ± 0.10 and 0.36 ± 0.01 dS/m, respectively. Organic matter (OM), Nt, and available P and K (Pav and Kav) contents were 3.93 ± 0.10%, 2.45 ± 0.04 g/kg, 38.3 ± 2.9 mg/kg, and 0.79 ± 0.03 g/kg, respectively. In addition, analyses revealed that the soil contained concentrations of heavy metals exceeding the maximum permissible limits established by Ecuadorian soil quality regulations. In particular, elevated levels of Cu (>25 mg/kg), Zn (>60 mg/kg), and Ni (>19 mg/kg) were detected, indicating contamination [34]. This situation justifies selecting this edaphic material as an experimental model to evaluate remediation strategies in soil contaminated with heavy metals from volcanic ash. The detailed results of these analyses are presented in Table S3.

Five treatments were established in a completely randomized design with 18 replicates per treatment (*n* = 90). They were placed in plastic pots 30 cm high and 30 cm in diameter. These treatments included: control without amendment (C), inorganic fertilization (100 kg N/ha; 50 kg P_2_O_5_/ha and 250 kg K_2_O/ha) (IF), and the three composts obtained from the composting experiment (17 t/ha; dry weight) C1F (Compost 1 without cheese whey), C2F (Compost 2 with whey diluted 1:2; *v*:*v*), and C3F (Compost 3 with undiluted whey). The amendments and inorganic fertilizers were incorporated 15 days before planting to facilitate their stabilization. Manual irrigation with a sprayer was used during this period to avoid desiccation and leaching. Subsequently, 18 uniform lettuce (*Lactuca sativa* var. Coolguard) seedlings were transplanted per treatment group and harvested 70 days later. Irrigation was carried out twice a week with tap water, maintaining soil moisture between 60 and 70%, which is optimal for crop development. Soil sampling was performed before planting (S1) and after harvest (S2). Large roots were removed from the soil samples before they were air-dried and sieved at 2 mm. The lettuce in each pot was cut after 70 days of growth, weighed to determine the fresh aboveground biomass/pot, and then dried at 60 °C for 48 h. Dry samples were ground and sieved to 0.5 mm for chemical analysis. All determinations in soil and vegetable samples were performed in triplicate.

### 2.3. Analytical Methods

In samples taken from the initial solid wastes for composting and throughout the composting process, the electrical conductivity (EC) and pH were determined in a 1:10 (*w*/*v*) aqueous extract. The dry matter content was determined gravimetrically after 12 h at 105 °C. The organic matter (OM) was calculated from the loss-on-ignition after 24 h at 430 °C. Automatic microanalysis was used to analyze Nt and Corg. The cation exchange capacity (CEC) was determined by completely replacing the exchangeable cations present in the organic materials with Ba^2+^, by saturating these materials with an excess of BaCl_2_-triethanolamine at pH 8.1, followed by gravimetric analysis. The modified Folin–Ciocalteu method was used to measure the water-soluble polyphenols in 1:20 (*w*/*v*) water extracts. To determine the germination index (GI), cress seeds (*Lepidium sativum* L.) were incubated in Petri dishes with aqueous extracts from samples taken at various stages of the composting process. The germination and root elongation results were expressed as percentages of the control (using deionized water instead of the extract). In the HNO_3_/HClO_4_ mineralization extract, P was determined colorimetrically as molybdovanadate phosphoric acid, while macro and micronutrients and heavy metals were analyzed by inductively coupled plasma mass spectrophotometry. For more detailed description, see the work by Idrovo-Novillo et al. [25]. The OM loss from each pile during composting was calculated according to Paredes et al. [35].

The physicochemical and chemical parameters analyzed in cheese whey (pH, EC, biochemical oxygen demand (BOD_5_), chemical oxygen demand (COD), total and suspended solids (TS and SS, respectively), and macronutrients) were measured according to the techniques detailed by Ramos-Romero et al. [23].

The pH and electrical conductivity (EC) of the soil samples were measured in 1:2.5 and 1:5 soil:water (*w*/*v*) extracts, respectively. Soil particle size analysis was performed by the Bouyoucos densimeter method, and oxidizable organic carbon was determined by the modified Walkley and Black method. The soil OM content was estimated by multiplying the percentage of organic carbon by the Van Bemmelen coefficient (1.724). To calculate the percentage of organic carbon, the percentage of oxidizable organic carbon was multiplied by 1.29, a recovery factor that includes the percentage of non-oxidizable organic carbon in the conditions of the technique used. Nt was determined by the Kjeldahl method. Available P (Pav) was analyzed colorimetrically by the method of Olsen. Extracts obtained with 1 N ammonium acetate were used to determine the soil available K (Kav) contents by inductively coupled plasma mass spectrophotometry. The available heavy metal concentrations were measured in a DTPA extract by inductively coupled plasma mass spectrophotometry. The mineral composition of the plants was determined on dried samples after HNO_3_-HClO_4_ digestion. P was analyzed by the colorimetric method of Kitson and Mellon, K and heavy metals by inductively coupled plasma mass spectrophotometry, and N by automatic microanalysis. For a more detailed description, see the work by Paredes et al. [36].

Finally, the bioaccumulation coefficient (BAC) of heavy metals was calculated to estimate the capacity of the tested crop to accumulate these potentially toxic soil elements in the edible parts of the plant and thus assess the potential risk to human health and food safety. To do this, the following equation was used [6]:

BAC = [total metal concentration (mg/kg)]_aboveground biomass_/[total metal concentration (mg/kg)]_soil_

### 2.4. Statistical Methods

Regarding the statistical methods used, the standard deviation was determined for the mean values of the parameters analyzed in the initial solid waste samples for composting, the cheese whey sample, the samples taken throughout the composting process, and the initial soil sample. The least significant differences (LSD) were calculated at the *p* < 0.05 significance level to determine the significant differences in the mean values of each parameter analyzed during the composting process. The OM loss during the composting of each pile was adjusted to the following first-order kinetic function [37]:

OM loss (%) = A (1 – e^−kt^)

in which A is the maximum degradation of OM (%), k is the rate constant (d^−1^), and t is the composting time (d). The values of adjusted R-squared (adj R^2^), standard error of estimation (SEE), and F were used to fit the curve of the experimental data on OM loss to the function and show the statistical significance of the curve fit. Furthermore, differences in agronomic value among the composts were determined by a one-way analysis of variance (ANOVA) with *p* < 0.05, and Tukey’s test was used to separate the mean values.

For the parameters determined in the soil samples, two variables were established: treatment and sampling. For the parameters measured in plant material samples, only the treatment variable was established. A one-way analysis of variance (ANOVA) was performed to determine significant differences in the data due to these variables. In addition, Tukey’s post hoc test was used to separate treatments and sampling means.

All statistical analyses of the data were performed using the IBM SPSS 27.0 statistical software package, except for the adjustment of the OM loss of the piles during the composting process to a first-order kinetic equation, which was performed using SigmaPlot 14.5.

## 3. Results and Discussion

### 3.1. Composting Process

**Figure 1:**
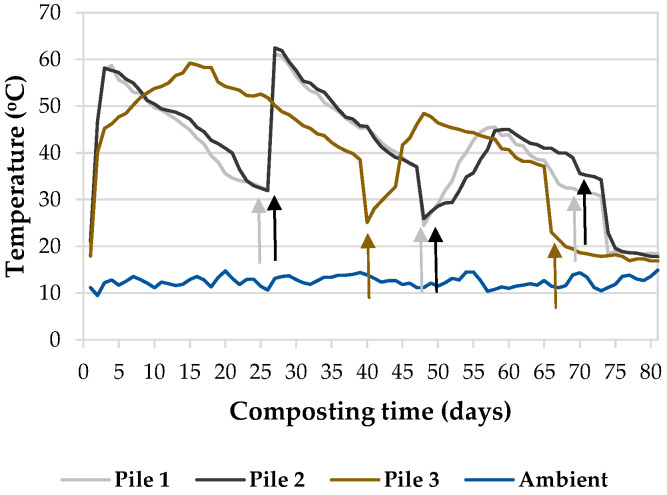
Temperature dynamics throughout the composting process (*n* = 5). The arrows indicate the days on which turnings were performed (Pile 1 = 60% maize waste + 5% bean waste + 35% cow dung, Pile 2 = 60% maize waste + 5% bean waste + 35% cow dung + diluted cheese whey, Pile 3 = 60% maize waste + 5% bean waste + 35% cow dung + undiluted cheese whey).

Temperature is a parameter that must be controlled during composting, as it indicates the activity of microorganisms. Thermophilic temperatures (>40 °C) were reached in the first 72 h in all the piles (Figure 1), probably due to the initial microbial decomposition of easily degradable compounds present in the pile without whey (pile 1) and in piles 2 and 3 with this liquid residue. Piles 1 and 2 maintained thermophilic conditions for 16 and 20 days, respectively, until the first turning. Pile 3 prolonged this phase to 37 days, possibly due to the added undiluted whey, which provided a significant amount of easily degradable carbon and nitrogen compounds. A prolonged thermophilic phase with increasing additions of different doses of whey was also observed by Şahin et al. [30] during the co-composting of rice husk with chicken manure or sewage sludge. When the temperature of the piles fell below 40 °C, they were turned. After the first turnings, the temperature rose due to the presence of oxygen, necessary for the aerobic degradation of organic matter and undecomposed materials, which became accessible with the homogenization of the waste mixture during turning. Piles 1 and 2 maintained thermophilic conditions for about 18 days after the first turning and for 10–12 days after the second turning. After the third turning, no increase in the temperature of these piles above thermophilic values was observed. However, in pile 3, thermophilic temperatures were observed for only 16 days after the first turning. After the second turning, temperatures decreased to values close to ambient. The average maximum temperatures recorded in piles 1 and 2 (61.1 °C and 62.5 °C, respectively) were higher than that observed in pile 3 (59.2 °C). All this indicates that microbial activity slowed down more rapidly during the composting process in pile 3, which can be attributed to the greater accumulation of salts in this pile due to the added undiluted whey. Liu et al. [38] observed that under extreme conditions of high salinity, microbial groups become specialized, causing microorganisms to move to narrower niches with less microbial diversity, thereby affecting the microbiological activity of the composting process.

**Figure 2:**
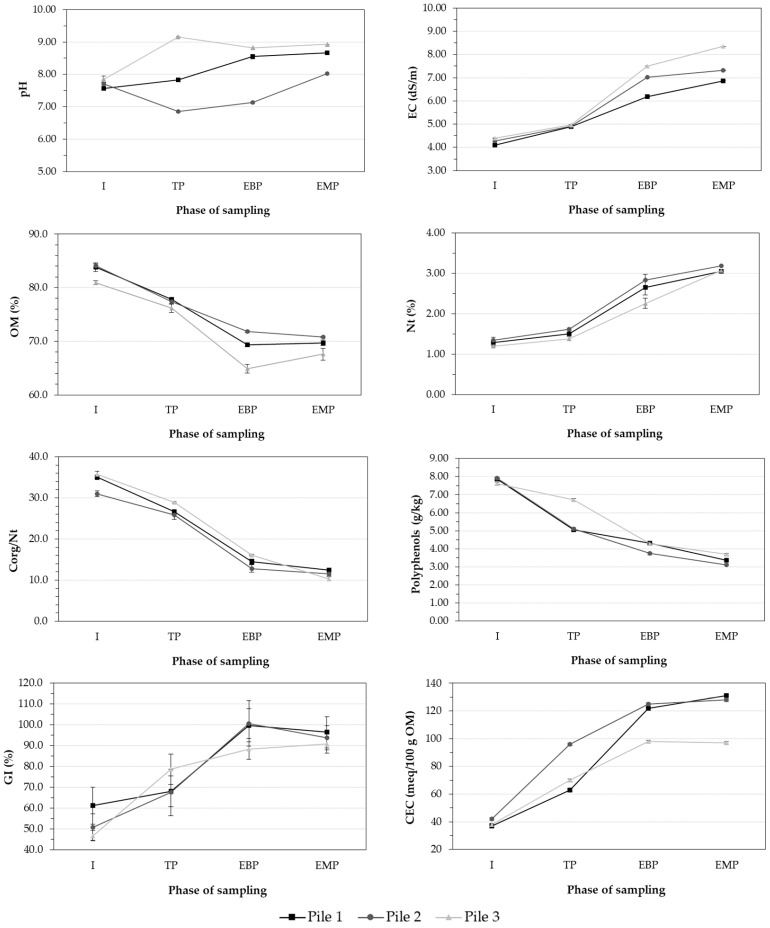
Evolution of the main parameters during composting (dry weight basis) (*n* = 1). Error bars represent the standard error of the mean. (Pile 1 = 60% maize waste + 5% bean waste + 35% cow dung, Pile 2 = 60% maize waste + 5% bean waste + 35% cow dung + diluted cheese whey, Pile 3 = 60% maize waste + 5% bean waste + 35% cow dung + undiluted cheese whey, I: initial phase, TP: thermophilic phase, EBP: end of bio-oxidative phase, EMP: end of maturation phase, EC: electrical conductivity, OM: organic matter, Corg: total organic carbon, Nt: total nitrogen, GI: germination index, CEC: cation exchange capacity).

In Figure 2, the initial pH values of piles 1, 2, and 3 (7.56, 7.70, and 7.85, respectively) fell within the optimal composting range (5.5–8), as reported by Bernal et al. [32]. Throughout the composting process, the piles showed fluctuations in pH. The increases in this parameter could be caused by the degradation of acidic compounds and the mineralization of nitrogenous compounds into ammonia [32]. The reduction in pH during the thermophilic phase could be attributed to the production of organic acids and CO_2_ dissolution generated by microorganisms during OM aerobic degradation [39]. However, the reduction in pH observed in pile 3 at the end of the bio-oxidative and maturity phases compared with the thermophilic phase was probably due to acidification produced by ammonium nitrification generated during the degradation of organic nitrogen [40]. An increase in electrical conductivity (EC) was observed, driven by the accumulation of inorganic salts resulting from organic matter mineralization and the concentration effect associated with weight loss in the pile [40]. In piles 2 and 3, this increased salinity was also due to the salt added from the whey, as evidenced by the higher final EC value in pile 3, with undiluted whey.

The degradation of organic matter throughout the composting process was reflected by the notable reduction in OM content observed in all piles. The reduction in this parameter ranged from 83.3%, 84.1%, and 80.9% to 69.7%, 70.8%, and 67.6% in piles 1, 2, and 3, respectively (Figure 2).

OM loss during composting was adjusted to the first-order kinetic model detailed in Section 2.4. with the parameters shown in Table 1. All piles showed a good fit of the experimental data on OM loss to the first-order kinetic equation, as indicated by the values obtained for F, R^2^ adj, and the standard error of estimation, and all equations were significant (*p* < 0.001 or *p* < 0.05). However, the results for piles 1 and 2 fit this equation better than those for pile 3, as demonstrated by their lower F and RMS and higher SEE values. Adding whey did not greatly affect the maximum OM degradation (A) compared with the control pile (pile 1). However, applying this liquid waste did have a significant effect on the rate of OM degradation (A × k), which was lower in pile 3. This could be due to the slowdown in microbial activity caused by the greater salt accumulation observed in this pile, as mentioned above.

Nt content showed a significant increase in all the piles during composting (Figure 2), possibly because of the concentration effect generated by the loss of composted material mass. This phenomenon was also described in Alfonzo et al.’s study [28], which evaluated the co-composting of grape pomace, green herbaceous crop, and pruning residues with and without deproteinized whey. In the three waste mixtures, the initial Corg/Nt ratio values were within or very close to the optimal range for proper composting development (Corg/Nt = 25–35). OM degradation and, with it, Corg and an increase in Nt throughout the composting process caused the Corg/Nt ratio to decrease, especially during the biooxidative stage. Subsequently, this ratio stabilized and, after one month of maturation, reached final values of 12.4, 11.5, and 10.3 in piles 1, 2, and 3, respectively, indicating the maturity and stability of the compost obtained. This decrease in the Corg/Nt ratio was also observed by Alfonzo et al. [28] and Şahin et al. [30] in different composting experiments using cheese whey.

Plant residues are characterized by their high polyphenolic content, which plants synthesize as a defense against phytopathogens and in response to abiotic stress caused by extreme radiation and climate conditions [41]. These compounds have also been linked to the phytotoxicity of composted plant materials, as they negatively affect seed germination and radicle emergence, with an increase in GI observed as polyphenolic substances degrade [21,24,25]. In this study, the reduction in soluble polyphenols during composting was between 52% and 61%, with pile 2 showing the greatest degradation of these compounds (Figure 2). However, all the piles exhibited a significant increase in GI throughout the composting process, with values of this index > 90% in mature composts, indicating a notable decrease in the abundance of phytotoxic compounds, such as water-soluble phenols, low-molecular-weight fatty acids, and ammonia [42]. Thus, adding whey did not affect the reduction in phytotoxicity in the materials to be composted. This was also observed by Michalopoulos et al. [26], who obtained a final compost free of phytotoxic substances by co-composting the solid fraction separately from a mixture of different manures and cheese whey.

The CEC is a widely used indicator of the evolution of the OM humification process during composting. Throughout this process, there is a gradual increase in negatively charged functional groups, such as carboxylic and/or hydroxyphenolic groups, which leads to an increase in these parameters [43]. In the piles studied, OM humification was confirmed by the rapid increase in this parameter during composting, with the highest values observed in mature samples, ranging from 97 to 131 meq/100 g OM (Figure 2).

Table 2 presents the main characteristics of the final composts. These composts presented alkaline pH values, which were significantly higher in composts 1 and 3 (8.67 and 8.93, respectively). The EC values of the composts that received whey (composts 2 and 3) were higher than those of compost 1. The compost watered with undiluted whey had the highest salinity level (compost 3). All composts exceeded the pH and EC values established by the US Composting Council [44] as suitable for different agricultural applications and field conditions. However, this did not negatively affect the GI, as the values were very high (GI > 90%) and above the minimum limit established by Zucconi et al. [42] (GI > 50%), indicating that the compost is mature and free of phytotoxicity. No significant differences in OM content were found among the composts, with OM contents above the range indicated by the American guidelines for agricultural compost use (OM = 50–60% [44]). The maturity of the composts and the humification of their OM were confirmed by their Corg/Nt ratios < 20, their CEC/Corg ratios > 1.9 meq/g Corg, and their CEC values > 67 meq/100 g OM [43,45]. The macronutrient contents were similar among the composts obtained, with values of 30.5–31.9 g/kg, 10.1–10.7 g/kg, and 31.3–32.0 g/kg for Nt, P, and K, respectively. The Nt and P contents were above the minimum values referenced by the US Composting Council [44] for different agricultural compost applications (Nt ≥ 10 g/kg and *p* ≥ 10 g/kg), showing the outstanding fertilizing capacity of the obtained composts. Adding undiluted whey resulted in a compost with significantly higher micronutrient contents, except for Zn, which was found in higher concentrations in compost 1, moistened with water. Regarding heavy metals, the compost without added whey (compost 1) had the highest content of many of these elements. However, all composts showed heavy metal concentrations well below the maximum limits established by the US Composting Council [44], indicating a low risk of toxicity in their agricultural use.

### 3.2. Effects of Compost on Soil Properties

**Figure 3:**
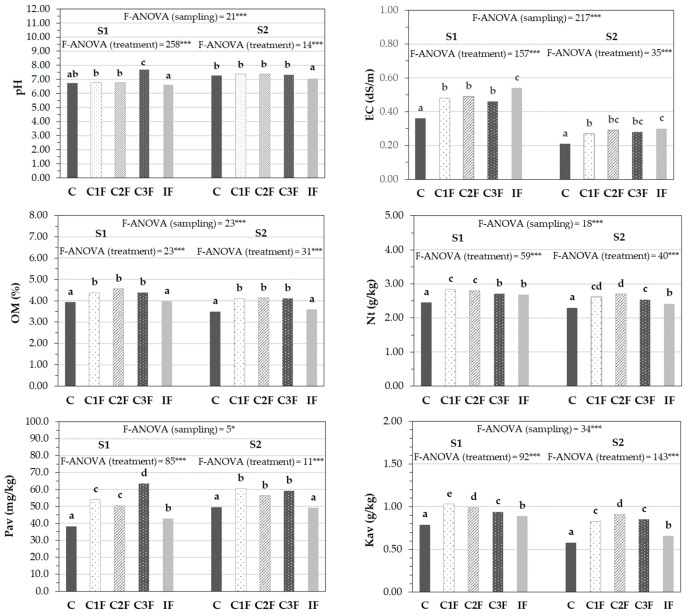
Evolution of soil agronomic parameters during lettuce cultivation (dry weight basis) (*n* = 18). (EC: electrical conductivity, OM: organic matter, Nt: total nitrogen, Pav: available P, Kav: available K, C: control without amendment, C1F: compost 1 without cheese whey, C2F: compost 2 with whey diluted 1:2 (*v*:*v*), C3F: compost 3 with undiluted whey, IF: inorganic fertilization, S1: before cultivation; S2: after harvesting the lettuce. *** and *: significant at *p* < 0.001 and 0.05, respectively. Mean values in columns followed by the same letter are not statistically different according to Tukey’s b test at *p* < 0.05).

The soil’s agronomic properties were directly influenced by the treatment used. As shown in Figure 3, the application of composts 1 and 2 and inorganic fertilizer did not significantly affect the initial pH of the control soil (C), with treatment C3F being the only one that significantly increased this parameter compared with C. This initial increase in soil pH is beneficial for the bioremediation of soils contaminated with heavy metals, as several authors have found that increased pH following compost application reduces the bioavailability of most heavy metals in soil [15,16,17,18]. Throughout the lettuce cultivation period, soil pH increased in most of the treatments, reaching values statistically similar to the control only in soils with organic amendments after harvest.

On the other hand, soils that received organic amendments (C1F, C2F, C3F) and inorganic fertilizer (IF) showed higher EC values in the initial stage of the experiment (S1) than the control treatment (C) (Figure 3). This increase was mainly attributed to the higher concentration of soluble salts provided by compost and mineral fertilizers. However, in none of the treatments did salinity reach levels that represented a risk to the development of the lettuce crop, since the values recorded remained well below the sensitivity threshold of the crop (EC < 1.30–1.75 dS/m [46]). The higher soil salinity with organic or inorganic fertilization persisted until after the lettuce harvest (S2). During crop development, EC decreased in most treatments, which could be explained by the active absorption of nutrients by the plants.

Adding compost to the soil produced a significant increase in OM of 10.9–15.4% and 15.5–16.3% compared with the average value of this parameter in treatments C and IF in the initial (S1) and final (S2) samples, respectively, as shown in Figure 3. Other authors have observed this positive effect in studies on the bioremediation of heavy metal-contaminated soils through compost application. They note that increases in soil OM can reduce the bioavailability of heavy metals such as Pb, Cu, or Cd by forming stable complexes with OM or through their precipitation with phosphate compounds added to the compost [15,16,18]. A slight decrease in OM was recorded between S1 and S2 for all the treatments. This could be due to the mineralization process in the soil mediated by microbial biomass. This evolution has also been observed in other research in Ecuador during compost application to Inceptisol soils for cut rose [47] and alfalfa [48] production.

Before planting, Nt, Pav, and Kav concentrations were generally higher in soils amended with compost than in the control soil or in soils amended with inorganic fertilizers (Figure 3), reflecting the remarkable fertilizing capacity of the composts used. This higher fertilizing capacity of the organic amendment treatments persisted throughout the experimental period, as soil macronutrient concentrations after harvest (S2) were significantly higher in C1F, C2F, and C3F than in the IF or C treatments. Thus, with compost application, the soil’s Nt, Kav, and Pav contents increased by factors of up to 1.1, 1.6, and 1.2, respectively, at the end of the experiment relative to their initial values, with the increases greater with C2F. Zeng et al. [17] also observed that applying 10% chicken manure compost increased the concentrations of soil macronutrients such as N and P, compared with an unamended control soil in rice fields contaminated with heavy metals. This demonstrates the important role of compost in improving the fertility of this type of contaminated soil. Throughout the lettuce growing cycle, Nt and Kav contents decreased, which could be attributed to the active nutrient uptake by the plants. However, Pav contents were significantly higher in S2 soils than in S1 soils in most treatments, suggesting that organic phosphorus likely mineralized gradually and compensated for losses due to chemical precipitation and crop uptake [49].

**Figure 4:**
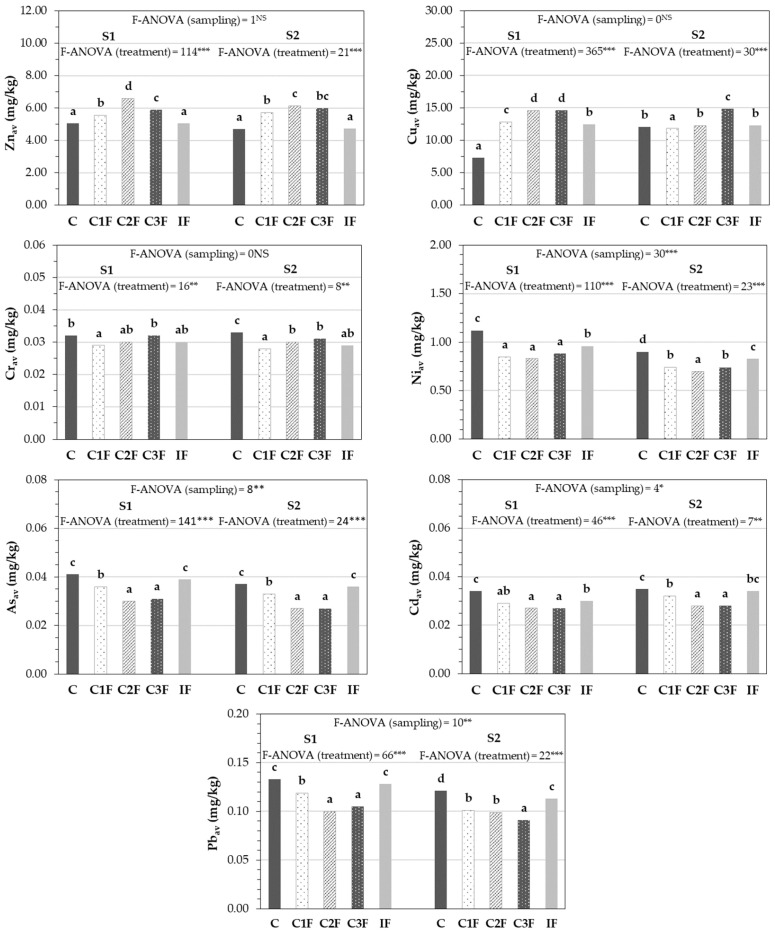
Evolution of the concentration of available heavy metals in the soil during lettuce cultivation (dry weight basis) (*n* = 18). (av: available, C: control without amendment, C1F: compost 1 without cheese whey, C2F: compost 2 with whey diluted 1:2 (*v*:*v*), C3F: compost 3 with undiluted whey, IF: inorganic fertilization, S1: before cultivation, S2: after harvesting the lettuce. ***, **, * and ^NS^: significant at *p* < 0.001, *p* < 0.01, *p* < 0.05, and not significant, respectively. Mean values in columns followed by the same letter are not statistically different according to Tukey’s b test at *p* < 0.05).

The impact of treatments and sampling on the availability of the heavy metals Zn, Cu, Ni, Cr, As, Cd, and Pb was studied, even though only the first three exceeded contamination limits (Table S3). This assessment is justified given that the phytotoxic risk in contaminated soils depends on the bioavailable fraction, which does not always correlate with the total concentration. Figure 4 shows that applying compost to the soil significantly increased the available Zn concentration compared with control soils and inorganic fertilization in the initial samples (S1). The concentration of available Cu at the start of the experiment was higher in all soils with organic or inorganic fertilization than in C, especially in treatments C2F and C3F. After the lettuce harvest, soils C1F, C2F, and C3F maintained the highest levels of available Zn, whereas this behavior was observed only in soils with C3F for Cu. Zeng et al. [17] also found that applying compost made from chicken manure at a rate of 10% to rice-growing soil increased the content of various heavy metals, including Zn and Cu, in the most mobile forms (exchangeable and carbonate-bound) compared with the control soil without amendment. This prevents the negative implications that the long-term use of this type of compost could have on the accumulation of these elements. Slight differences were found in available Cr concentrations among the different treatments tested in the S1 soil samples. However, at the end of the cultivation period, the content of this heavy metal was statistically lower in all soils with organic or inorganic fertilization (C1F, C2F, C3F, or IF) compared with the control soil. Applying compost to the soil significantly reduced available Ni concentrations at the beginning and end of the cultivation period (S1 and S2, respectively) compared with treatments C and IF, with the control treatment showing the highest levels of this heavy metal in S1 and S2. The reduction in Ni bioavailability with compost application could be related to Ni being absorbed into its OM, which contains functional groups with high cation exchange capacity (carboxyl and hydroxyl groups) [19]. Throughout the experimental period, assimilable As content was also statistically lower in soils with organic amendments, especially in treatments C2F and C3F, compared with soils with inorganic fertilization and the control. In this study, it was not observed that an increase in soil pH and the presence of negatively charged functional groups at the cation exchange sites produced by adding compost favored As bioavailability through its desorption of As-oxyanions, as other authors have found [20]. Furthermore, compost use reduced the bioavailable Cd and Pb contents of the soil before lettuce planting (S1) and after harvest (S2). In the case of Cd, the C2F and C3F treatments were most effective at immobilizing this potentially toxic element compared with the C and IF treatments, while the three composts tested reduced Pb bioavailability compared with soils with mineral fertilizers and without fertilization (C). Various authors have reported the importance of applying compost to the soil as a strategy for reducing Cd and Pb mobility. The bioavailability of these heavy metals is reduced due to the increase in soil pH with compost application, reducing the solubility of these elements, the formation of stable Cd or Pb-organic matter complexes, and the precipitation of these heavy metals with phosphorus compounds [15,18,50]. In relation to the evolution of available heavy metals in the soil throughout the lettuce cultivation experiment, no significant differences were found (or they were very slight) in the contents of Zn, Cu, Cr, and Cd between samples S1 and S2, indicating a gradual change in the state of these elements to a bioavailable form, thus compensating for the loss due to absorption by the crop. However, significant reductions in Ni, As, and Pb concentrations were found over time, probably due to their immobilization through the mechanisms mentioned above when compost was added to the soil, and these elements were taken up by the lettuce plants.

### 3.3. Effects of Compost on the Plant Yield, Nutritional Composition, and Heavy Metal Accumulation of Lettuce

**Figure 5:**
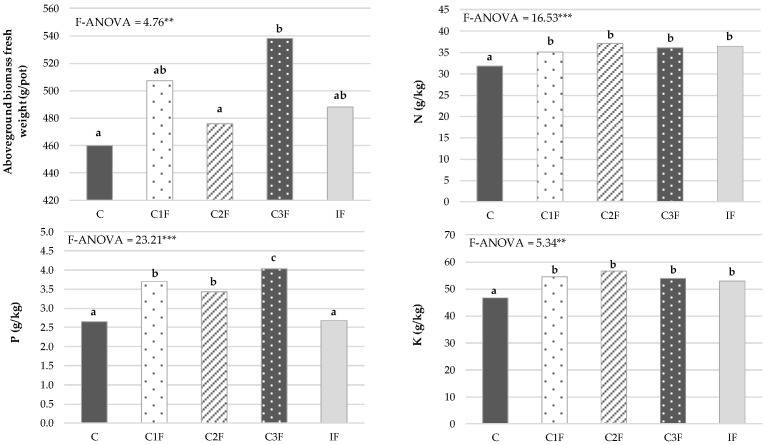
Yield of lettuce and its macronutrient concentrations (dry weight basis) (*n* = 18). C: control without amendment, C1F: compost 1 without cheese whey, C2F: compost 2 with whey diluted 1:2 (*v*:*v*), C3F: compost 3 with undiluted whey, IF: inorganic fertilization. *** and **: significant at *p* < 0.001 and 0.01, respectively. Mean values in columns followed by the same letter are not statistically different according to Tukey’s b test at *p* < 0.05.

Figure 5 shows that the aboveground fresh weight biomass of lettuce was statistically higher in plants grown with the C3F treatment, while lettuce with the lowest weight was found in treatments C and C2F. Soils with treatments C1F and IF had intermediate lettuce production compared with the previous treatments, with no significant differences between them. These results indicate the high fertilizing potential of composts 1 and 3 and their ability to promote lettuce growth in soils contaminated with different heavy metals. Other authors have also found that compost use increases plant growth under heavy metal stress, such as winter wheat (*Triticum aestivum* L.) in soils contaminated with Cd [18], hybrid maize (*Zea mays* L.) in soils contaminated with Ni [19], maize (*Zea mays* L.) in soils contaminated with As [20], and annual grass (*Oenothera picensis*) in soils contaminated with Cu [16]. In the case of lettuce grown on compost 2 (C2F), this effect was not found, with very low plant growth that was statistically equivalent to that of the control soil, probably due to greater As assimilation by the plants in this treatment, as will be seen below.

Regarding the macronutrient content in lettuce leaves, the treatments with compost (C1F, C2F, and C3F) and inorganic fertilizer (IF) caused significant increases in leaf concentrations of N and K compared with the control treatment (C) (Figure 5). No significant differences were found in the foliar contents of these macronutrients between the treatments with compost and mineral fertilization. These results indicate the remarkable ability of these composts to improve the fertility of the soils contaminated with heavy metals from volcanic ash. Moreover, the compost treatments showed statistically higher increases in P concentration in lettuce leaves than the C and IF treatments, which produced plants with the lowest P concentrations. Mehmood et al. [20] also observed that the phosphorus concentration in maize shoots was higher in the compost treatments than in the unamended sandy loam control soil contaminated with As. It is noteworthy that the plants with the highest P content were grown on C3F, which may have contributed to the higher lettuce weight in this treatment, since phosphorus plays an important role in plant metabolism and participates significantly in photosynthesis, respiration, and cell division [51].

**Figure 6:**
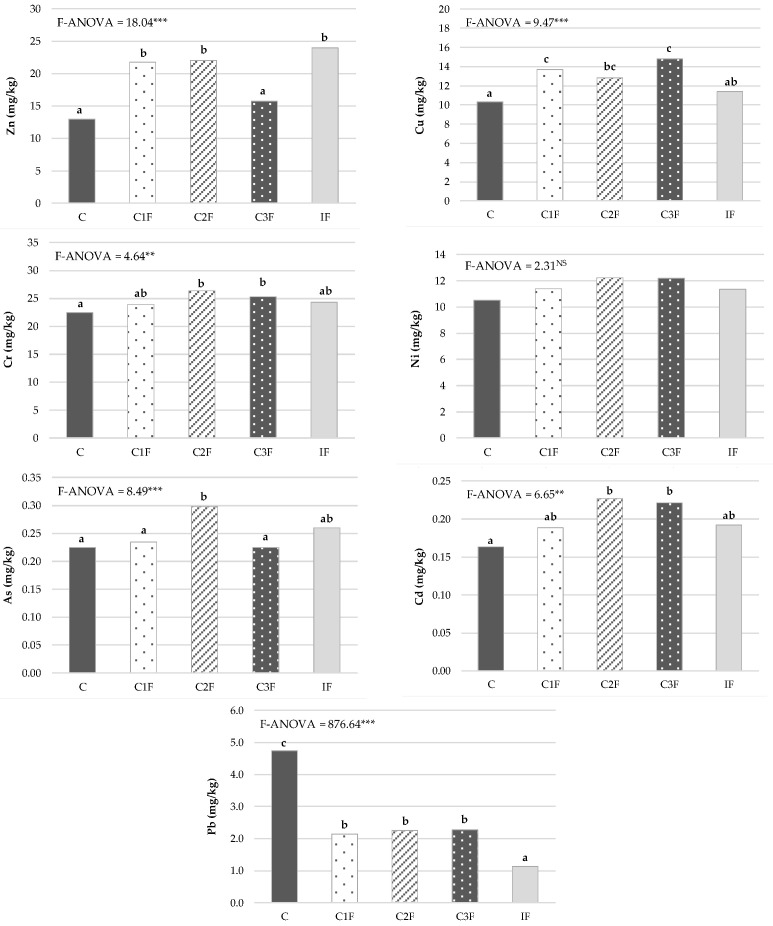
Heavy metal concentrations of lettuce (dry weight basis) (*n* = 18). C: control without amendment, C1F: compost 1 without cheese whey, C2F: compost 2 with whey diluted 1:2 (*v*:*v*), C3F: compost 3 with undiluted whey, IF: inorganic fertilization. ***, ** and ^NS^: significant at *p* < 0.001, *p* < 0.01, and not significant, respectively. Mean values in columns followed by the same letter are not statistically different according to Tukey’s b test at *p* < 0.05.

The heavy metal content in plant tissue was directly influenced by the type of treatment used, as shown in Figure 6. The foliar Zn concentration was significantly higher in lettuce from treatments C1F, C2F, and IF, ranging from 22 to 24 mg/kg (dry basis). However, treatment with C3F reduced the presence of this element in the plant to levels statistically similar to those observed in the control treatment. None of the lettuce produced in this study had a Zn content above toxic levels for plants (Zn = 100–500 mg/kg (dry basis) [52]), and the estimated BAC values for this plant showed that Zn was not accumulating (BAC < 1 in all the treatments tested) (Table 3). This result demonstrates the low toxicity of Zn in the soil studied, despite total Zn concentrations exceeding the maximum limits established by the Ecuadorian soil quality standard [34] (Table S3). This indicates that the possible negative effects of heavy metals on crops in contaminated soils depend on their bioavailable concentrations, which, in many cases, are not correlated with total concentration values [53].

Cu concentrations in the leaf tissues of *Lactuca sativa* ranged from 10 to 15 mg/kg (dry basis), with significant differences among treatments (*p* < 0.001). Compost treatments generally increased the concentration of this element in the plant compared with IF and C (Figure 6). Zeng et al. [20] and Rehman et al. [19] also observed an increase in Cu concentration in rice and straw grown in soil contaminated with heavy metals from mining activities and in shoots and roots of maize seedlings grown in soil contaminated with Ni, respectively, when comparing compost treatments with the control soil without amendment. This increase in Cu in crops could be related to greater nutrient availability from compost application or to a synergistic effect that improved the absorption of this heavy metal [20]. In this study, all values recorded for Cu in plants were within the critical toxic concentration range for Cu in lettuce (Cu = 8–23 mg/kg (dry basis)) according to Oorts [54]. However, in none of the treatments tested was a BAC value > 1 found in lettuce. This indicates that there is no Cu accumulation in plants (Table 3) despite the high Cu content present in this soil, which exceeded the minimum threshold established by the Ecuadorian soil quality standard [34] in its total concentration (Table S3).

Concentrations of Cr in plant material were statistically higher in lettuce from treatments with composts 2 and 3 (C2F and C3F) and lower in plants from the control treatment, with values intermediate between the former in the tissues of C1F and IF plants (Figure 6). The greatest increase in leaf Cr concentration in lettuce with compost use compared with a control without amendment was also observed by Medyńska-Juraszek et al. [50]. They tested different amendments, such as compost, biochar, and their mixtures, as immobilizing agents to reduce the uptake of heavy metals by green leafy vegetables grown in soil with high concentrations of heavy metals. The foliar values of this heavy metal ranged between 22 and 26 mg/kg (dry basis), well above the range of critical Cr concentrations in leaves indicated by Gonnelli and Renella [55] (1–10 mg/kg (dry basis)). Furthermore, across all treatments, the BAC values for this potentially toxic element in lettuce were very close to or above the limit value, indicating bioaccumulation of a heavy metal in a crop (BAC > 1) (Table 3). These results show the high potential for Cr bioaccumulation in edible tissues. Although Cr toxicity depends on its chemical form (Cr(VI) is the most toxic), these levels indicate a significant entry of this metal into the plant-soil system and justify monitoring its translocation, especially in crops destined for fresh consumption, such as lettuce.

No significant differences were found in Ni concentrations in lettuce leaves among the treatments studied, with the concentration of this heavy metal in plant material ranging from 11 to 12 mg/kg (dry basis) (Figure 6). Although the available information on Ni content in leafy vegetables is limited, with regard to Ni toxicity, critical toxicity levels are generally >10 mg/kg (dry basis) in sensitive species and >50 mg/kg (dry basis) in moderately tolerant species [55]. Therefore, Ni levels in the lettuce in this experiment slightly exceeded the thresholds associated with this element’s toxicity in sensitive species. BAC values for this element were <1 in all plants analyzed, regardless of the treatment used (Table 3). These results indicate low Ni bioaccumulation in the tested crop, although the total Ni concentration in the soil was very high relative to the Ecuadorian soil quality standard [34] (Table S3).

For foliar As, values ranged from 0.23 to 0.30 mg/kg (dry basis), showing significant differences (*p* < 0.001) among plants grown under the different treatments (Figure 6). The plant material from the soil with C2F showed the highest content of this heavy metal, which contradicted the reduction in the availability of this heavy metal in the soil with the application of this compost (Figure 4). This higher As content in plants grown with C2F may have been responsible for the lower lettuce growth in this treatment compared with the other compost treatments (C1F and C3F) (Figure 5). Mehmood et al. [20] linked the phytotoxicity induced by increasing arsenic application in two types of soil with different textures to reduced growth in maize plants, attributing this effect to the possible malfunctioning of metabolic processes, such as respiration and photosynthesis, in plants subjected to As stress. The arsenic content of different foods is limited by the Codex Alimentarius Commission [56], but the limit for arsenic in leafy vegetables has not been established. Furthermore, the concentrations of this metalloid in plants depend largely on the species, cultivar, and ecotype. However, the As content in the lettuce in this study was within the range of As concentrations in plants grown in uncontaminated soils (0.009–1.5 mg kg (dry basis)) according to Wenzel [57]. Moreover, the BAC values for As in the lettuce in the study were well below the threshold for considering bioaccumulation of this metalloid in the plant (Table 3). Therefore, it could be considered that there is no high risk of As contamination in crops grown in the soil studied.

The Cd content in the cultivated lettuce ranged from 0.16 to 0.23 mg/kg (dry basis), with the lowest and highest contents of this element found in the control plants and those cultivated on composts 2 and 3, respectively (Figure 6). The Cd concentration in the lettuce in this experiment was very low relative to the limit established for leafy vegetables in the Codex Alimentarius Commission [56] (<0.2 mg/kg (fresh basis), considering that the average water content of the lettuce in the experiment was 93%, and its Cd content on a fresh basis ranged from 0.01 to 0.02 mg/kg). This result may be due to the ability of roots to sequester this metal in their vacuoles, thus reducing its translocation to the aerial parts of the plant. This could explain the low Cd concentration in the lettuce analyzed [58]. However, across all the treatments, a BAC value for this heavy metal in the plant above 1 was observed, indicating a high risk of Cd bioaccumulation in the plant’s edible parts (Table 3). Thus, compost use requires careful optimization before large-scale application due to the potential risk of Cd accumulation in plants grown in it, in order to prevent risks to human health and ensure food safety.

Regarding the Pb content in lettuce leaves, applying compost or mineral fertilizers reduced the foliar content of this heavy metal compared with plants grown in control soil without any fertilization (C) (Figure 6). In the compost treatments (C1F, C2F, and C3F), the presence of Pb in lettuce was reduced by an average of 53%, while in the IF treatment, this reduction was 76%. Medyńska-Juraszek et al. [50] also observed a notable reduction in leaf Pb concentration in lettuce grown with compost compared with plants grown in soil with high concentrations of heavy metals and without organic amendment. The foliar Pb contents in the lettuce in this study ranged from 1.1 to 4.7 mg/kg (dry basis), which were only above the limit established for leafy vegetables in the Codex Alimentarius Commission in the control treatment [56] (<0.3 mg/kg (fresh basis), considering that the average water content of the lettuce in the experiment was 93%, and its Pb content on a fresh basis ranged from 0.08 to 0.33 mg/kg). This indicates the effectiveness of applying compost or mineral fertilizers to reduce the accumulation of this heavy metal in the crop tested, although the bioaccumulation of this heavy metal in the plant was low in the soil studied (BAC of Pb < 1 in lettuce in all treatments) (Table 3).

In this study, no relationship was observed between the amount of heavy metal available in the soil and its accumulation in the plant. For most of the heavy metals studied, their mobility in the soil was reduced by compost application, especially for Ni, As, Cd, and Pb, compared with the IF and C treatments. However, the highest concentrations of some of these elements were obtained in lettuce grown with the composts used, and their BAC values were also higher, as was the case with As with C2F and Cd with C2F and C3F. This was also observed by Medyńska-Juraszek et al. [50] for Cd and Pb, which were bioaccumulated in greater quantities in green leafy vegetables grown in compost treatments, while compost application resulted in a notable immobilization of these elements in the soil. These authors indicated that this result could not be attributed to the soluble forms of both metals in the soil solution, but rather to changes in metal speciation or the reduction in soil pH amended with compost, which could influence metal absorption under the conditions of the soil studied.

## 4. Conclusions

Based on the results obtained, it can be concluded that co-composting agro-livestock waste with cheese whey produced composts containing stabilized organic matter, free of phytotoxins, and with an adequate degree of maturity. In addition, these composts had high levels of organic matter and macronutrients, demonstrating their remarkable fertilizing capacity The main effects of increasing the whey dose during composting were a slowdown in microbial activity and, consequently, a lower rate of organic matter degradation, and an increase in the salinity of the materials to be composted throughout the process; moreover, the application of undiluted cheese whey increased the content of most micronutrients in the final compost.

In addition, applying agro-livestock waste composts enriched with whey improved the fertility of volcanic-ash-affected soils by increasing macronutrient and organic matter contents, outperforming mineral fertilizers. Also, these organic amendments proved to be more effective in reducing the availability of heavy metals such as Ni, As, Cd, and Pb; however, they increased the availability of Zn and Cu. In addition, the use of compost with undiluted whey (C3F) resulted in higher lettuce yields than those obtained with inorganic fertilization, positioning this compost as a possible alternative to inorganic fertilizers for this crop in the contaminated soils studied. No correlation was observed between lower heavy metal availability in the soil and their accumulation in the plant, as the compost treatments did not consistently protect the lettuce from heavy metal uptake. The contaminated soils studied showed a high risk of Cr and Cd accumulation in the edible parts of lettuce, with composts unable to reduce this assimilability and even increasing it; similarly, the accumulation of As in plants grown with C2F contradicted the observed reduction in its availability in the soil. On the other hand, it is worth noting that the soils studied showed low toxicity for Cu, Zn, and Ni, despite their total concentrations exceeding the maximum limits established by the Ecuadorian soil quality standard. This finding confirmed that the potential negative effects of heavy metals on crops grown in contaminated soils depend on their bioavailable fraction, which, in many cases, bears no relation to total concentration values.

Therefore, co-composting the agricultural and livestock waste studied with cheese whey as a source of moisture can play an important role in the circular bioeconomy, with the use of the compost obtained in remediating soils contaminated with heavy metals from volcanic ash. However, the compost dosage and the crop to be treated must be optimized before large-scale use, since the application of this type of organic amendment proved insufficient to consistently mitigate the transfer of heavy metals to the crop. This work has initiated the study of using eco-technologies for contaminated soil remediation in the context of volcanic environments in Ecuador. The information obtained may be useful for decision-making by farmers’ associations, agricultural technicians, and local politicians to establish safety and monitoring limits to protect consumers from heavy metal contamination in agricultural production.

## Figures and Tables

**Table 1:** Values of the parameters of the first-order kinetic equation for the studied piles (Pile 1: 60% maize waste + 5% bean waste + 35% cow dung (water); Pile 2: 60% maize waste + 5% bean waste + 35% cow dung (diluted whey; 1:2 (*v*:*v*)) and Pile 3: 60% maize waste + 5% bean waste + 35% cow dung (undiluted whey)).

Piles	A (%)	k (Days^−1^)	A × k	R^2^ Adj	F	SEE
P1	58.5	0.0350	2.0475	0.9931	572.24 ***	2.07
P2	54.5	0.0418	2.2781	0.9993	5921.96 ***	0.61
P3	58.2	0.0235	1.3677	0.8990	26.69 *	8.71

A: maximum OM degradation, k: rate constant, R^2^ Adj: adjusted R-Squared, SEE: standard error of estimate. ***, *: significant at *p* < 0.001 and *p* < 0.05, respectively.

**Table 2:** The main characteristics of the final composts (dry weight basis) (Compost 1: 60% maize waste + 5% bean waste + 35% cow dung (water); Compost 2: 60% maize waste + 5% bean waste + 35% cow dung (diluted whey; 1:2 (*v*:*v*)) and Compost 3: 60% maize waste + 5% bean waste + 35% cow dung (undiluted whey)) (*n* = 1).

Parameters	Compost 1	Compost 2	Compost 3	F-ANOVA	US Guidelines ^a^
pH	8.67 b	8.02 a	8.93 c	36,443.7 ***	6.0–7.5
EC (dS/m)	6.87 a	7.31 b	8.35 c	98,183.2 ***	<5
OM (%)	69.7	70.8	67.6	5.1 ^NS^	50–60
Corg/Nt	12.4 b	11.5 ab	10.3 a	14.5 *	-
GI (%)	96.5	93.8	90.9	0.5 ^NS^	-
CEC (meq/100 g OM)	131 b	128 b	97 a	81.6 **	-
CEC/Corg (meq/g Corg)	2.42 b	2.47 b	2.07 a	30.1 **	-
Nt (g/kg)	30.5 a	31.9 b	30.7 a	58.9 **	≥10.0
P (g/kg)	10.6 b	10.7 b	10.1 a	34.7 **	≥10.0
K (g/kg)	31.5	31.3	32.0	0.3 ^NS^	-
Fe (mg/kg)	1978 b	1576 a	2550 c	1175.2 ***	-
Cu (mg/kg)	23 a	21 a	36 b	12.6 *	1500
Mn (mg/kg)	125 b	115 a	149 c	198.7 ***	-
Zn (mg/kg)	115 c	105 b	73 a	195.6 ***	2800
Ni (mg/kg)	16	10	12	2.0 ^NS^	420
Cr (mg/kg)	99 b	81 a	101 b	18.7 *	-
Cd (mg/kg)	0.40	0.29	0.55	8.9 ^NS^	39
Pb (mg/kg)	12.0 b	2.8 a	2.5 a	155.8 ***	300
As (mg/kg)	1.07 a	0.94 a	1.31 b	55.6 **	41
Hg (mg/kg)	<0.05	<0.05	<0.05	-	17
Se (mg/kg)	2.88 b	1.87 a	1.74 a	68.4 **	100

EC: electrical conductivity, OM: organic matter, Corg: total organic carbon, Nt: total nitrogen, GI: germination index, CEC: cation exchange capacity. ***, **, * and ^NS^: significant at *p* < 0.001, *p* < 0.01, *p* < 0.05, and not significant, respectively. Values in a row followed by the same letter are not statistically different according to Tukey’s b test at *p* < 0.05. ^a^ According to US Composting Council [44].

**Table 3:** Bioaccumulation coefficient (BAC) of heavy metals in lettuce. C: control without amendment, C1F: compost 1 without cheese whey, C2F: compost 2 with whey diluted 1:2 (*v*:*v*), C3F: compost 3 with undiluted whey, IF: inorganic fertilization.

Treatment	Zn	Cu	Cr	Ni	As	Cd	Pb
C	0.07	0.15	0.90	0.42	0.07	1.36	0.47
C1F	0.11	0.20	0.96	0.46	0.07	1.57	0.21
C2F	0.11	0.19	1.06	0.49	0.09	1.89	0.23
C3F	0.08	0.21	1.01	0.49	0.07	1.85	0.23
IF	0.12	0.17	0.97	0.45	0.08	1.60	0.11

The colors indicate the intensity of metal bioaccumulation: green chromatic scale, low bioaccumulation; yellow chromatic scale, medium; orange chromatic scale, high; red chromatic scale, very high bioaccumulation of given metal.

## Data Availability

All data and original contributions presented in this study are included in the article and the Supplementary Materials. Further inquiries can be directed to the corresponding author.

## Supplementary Materials

The following supporting information can be downloaded at: https://www.mdpi.com/article/10.3390/plants15101507/s1. Table S1. Characteristics of the initial solid materials: maize waste (MW), bean waste (BW), and cow dung (CD) (dry weight basis) (n = 1). Table S2. Physicochemical and chemical characterization of cheese whey used (n = 1). Table S3. Total heavy metal contents in the soil used (dry weight basis) (n = 1).
