# Supplementary material for: Production of Composts from Cheese Whey and Agro-Livestock and Their Valorization in Volcanic-Ash-Affected Soil Cultivated with Lactuca sativa L
Source: Plants (Basel). 2026 May 15;15(10):1507. doi: 10.3390/plants15101507 (PMC13210563; doi:10.3390/plants15101507)
Supplement: Supplementary file 1 [file plants-15-01507-s001.zip › plants-4241190-supplementary.pdf]

Production of composts from cheese whey and agro-livestock and their valorization in volcanic-ash-affected soil cultivated with *Lactuca sativa* L.

Steven Ramos-Romero [1], Irene Gavilanes-Terán [2], Julio Idrovo-Novillo [2], Sandra N. Escobar-Arrieta [2], María José Bermeo [2], Alessandro Idrovo-Gavilanes [3], Julio Idrovo-Gavilanes [4] and Concepción Paredes [5,*]

1. Faculty of Agricultural Industries and Environmental Sciences, Carchi State Polytechnic University, 040102 Tulcán, Carchi, Ecuador; steven.ramos@upec.edu.ec (S.R.-R.)
2. Faculty of Science, Higher Polytechnic School of Chimborazo, EC 060155, Riobamba, Chimborazo, Ecuador; irene.gavilanes@espoch.edu.ec (I.G.-T.); julio.idrovo@espoch.edu.ec (J.I.-N.); saescobar@espoch.edu.ec (S.N.E-A.); jose.bermeo@espoch.edu.ec (M.J.B.)
3. Faculty of Medicine and Health Sciences, Ghent University, Corneel Heymanslaan 10, 9000 Ghent, Belgium; alessandro.idrovo@ugent.be (A.I.-G.)
4. Institute for Research in Biomedicine of Lleida (IRBLleida), Av. Alcalde Rovira Roure, 80, 25198 Lleida, Spain; jidrovo@irblleida.cat (J.I.-G.)
5. Agri-Food and Agro-Environmental Research and Innovation Institute (CIAGRO-UMH), Miguel Hernandez University, EPS-Orihuela, ctra. Beniel km 3.2, 03312, Orihuela (Alicante), Spain

* Correspondence: c.paredes@umh.es (C.P.)

Supplemental material

Table S1. Characteristics of the initial solid materials: maize waste (MW), bean waste (BW), and cow dung (CD) (dry weight basis) (n =1).

Parameter	MW	BW	CD
pH	4.84 ± 0.02	4.96 ± 0.05	7.79 ± 0.06
EC (dS/m)	3.42 ± 0.35	5.52 ± 0.13	6.06 ± 0.10
OM (%)	91.3 ± 0.1	88.6 ± 0.1%	77.7 ± 0.9
Corg (%)	43.7 ± 0.2	41.1 ± 1.4	41.0 ± 0.2
Nt (%)	0.74 ± 0.04	3.32 ± 0.18	2.16 ± 0.16
Corg/Nt	59.1 ± 3.1	12.4 ± 0.3	19.0 ± 1.4
P (g/kg)	3.05 ± 0.07	3.85 ± 0.14	7.46 ± 0.20
K (g/kg)	15.8 ± 1.5	25.5 ± 0.9	10.2 ± 0.0
Fe (mg/kg)	688 ± 42	920 ± 33	1503 ± 36
Mn (mg/kg)	26.5 ± 2.1	44.5 ± 2.1	93.0 ± 1.4
Cu (mg/kg)	6.5 ± 0.7	12.0 ± 1.4	26.5 ± 0.7
Zn (mg/kg)	32 ± 2	61 ± 3	106 ± 6
Ni (mg/kg)	8 ± 0	5 ± 2	13 ± 3
Cr (mg/kg)	2.0 ± 0.1	3.1 ± 0.1	85.5 ± 6.2
Cd (mg/kg)	0.13 ± 0.01	0.10 ± 0.01	0.21 ± 0.03
Pb (mg/kg)	< 0.1	0.40 ± 0.06	1.10 ± 0.11
As (mg/kg)	0.20 ± 0.03	0.39 ± 0.06	0.50 ± 0.02

EC: electrical conductivity, OM: organic matter, Corg: total organic carbon, Nt: total nitrogen. Values reported as mean ± standard error.

Table S2. Physicochemical and chemical characterization of cheese whey used (n=1).

Parameter	Value
pH	6.77 ± 0.07
EC (dS/m)	5.03 ± 0.04
BOD_5 (mg O_2/L)	32,244 ± 300
COD (mg O_2/L)	66,000 ± 549
TS (mg/L)	73,400 ± 173
SS (mg/L)	3518 ± 3
N (g/L)	0.62 ± 0.01
P (g/L)	0.10 ± 0
K (mg/L)	2.94 ± 0.02
Fe (mg/L)	0.36 ± 0.01
Cu (mg/L)	< 0.1
Zn (mg/L)	0.71± 0.02
Ni (mg/L)	< 0.1
Cr (mg/L)	< 0.1
Cd (mg/L)	< 0.1
Pb (mg/L)	< 0.1

EC: electrical conductivity; BOD_5: biochemical oxygen demand; COD: chemical oxygen demand; TS: total solids; SS: suspended solids. Values reported as mean ± standard error.

Table S3. Total heavy metal contents in the soil used (dry weight basis) (n = 1).

Parameter	Value	Soil Quality Criteria of the Ecuadorian Standard[1]
Cu (mg/kg)	69 ± 1	25
Zn (mg/kg)	198 ± 1	60
Ni (mg/kg)	25 ± 1	19
Cr (mg/kg)	25 ± 4	54
Cd (mg/kg)	0.12 ± 0	0.5
Pb (mg/kg)	10 ± 1	19
As (mg/kg)	3.36 ± 0.17	12

[1]Ministerio del Ambiente de Ecuador [34]
